# A feedback-driven ventilation model for assessing airway secretions in mechanically ventilated patients

**DOI:** 10.3389/fphys.2025.1612501

**Published:** 2025-06-13

**Authors:** D. Vijay Anand, Manuel Teixeira Cabeleira, Claire Black, Vanessa Diaz-Zuccarini, Nicholas C. Ovenden

**Affiliations:** ^1^ Department of Mathematics, University College London, London, United Kingdom; ^2^ Department of Mechanical Engineering, University College London, London, United Kingdom; ^3^ Therapies and Rehabilitation, University College London Hospitals NHS Foundation Trust (UCLH), London, United Kingdom

**Keywords:** mechanical ventilation, airway clearance, secretion management, compartmental model, ventilator waveforms

## Abstract

**Introduction:**

A mechanistic compartmental model with a feedback-driven simulation framework was developed to investigate the impact of airway secretion accumulation and its removal on the respiratory dynamics of mechanically ventilated patients. Understanding these dynamics is essential for secretion management and improving respiratory care in the intensive care unit (ICU).

**Methods:**

The model simulates pressure support ventilation by incorporating airway resistances, lung and chest wall compliances, and patient effort via a dynamic respiratory muscle pressure term, enabling realistic modelling of patient-ventilator interaction. To validate the model, simulated waveforms were compared against clinical waveform recordings. Waveform features sensitive to secretion-related changes, as indicated by the model, were then extracted from the patient waveform recordings. The Wasserstein distance metric was used to quantify shifts in pre- and post-suction feature distributions, and unsupervised clustering was applied to identify distinct patient groups corresponding to low, medium, and high secretion levels.

**Results:**

The simulations revealed characteristic changes in ventilator waveforms associated with secretion accumulation, including reduced inspiratory flow and prolonged expiration. Analysis of patient data using clustering methods identified distinct groups corresponding to low, medium, and high levels of secretion. Further, we introduce a model-informed secretion index derived from the simulations and patient data, enabling non-invasive and continuous monitoring of secretion accumulation at the bedside.

**Conclusions:**

This study demonstrates the potential of physiology-informed, model-based approaches for real-time assessment of secretion accumulation in mechanically ventilated patients. The proposed framework supports personalized respiratory care by providing clinicians with data-driven insights into secretion accumulation, paving the way for more precise secretion management strategies in the ICU.

## 1 Introduction

Mechanical ventilation is initiated to support or take over a patient’s breathing when they are unable to independently sustain adequate ventilation. However, the introduction of an artificial airway in order to deliver mechanical ventilation results in impairment of the normal mechanisms underpinning airway clearance; airway humidification, mucocilliary escalator and cough. Retention of airway secretions is a common and serious problem in ventilated patients. Retained mucus narrows or occludes the airways (Roe et al., 2025; Mietto et al., 2014), causes respiratory distress, provides a growth medium for bacteria, and if extensive, leads to atelectasis, gas exchange impairment, and ventilator-acquired pneumonia (VAP). VAP is the most common nosocomial infection affecting patients in critical care and is associated with increased mortality and antibiotic use (Howroyd et al., 2024). Excess secretions increase the effort to breathe, making it harder for patients to transition to spontaneous breathing, complicating the weaning process and increasing the risk of extubation failure (Branson, 2007; Volpe et al., 2020; Goetz et al., 2022). Typically, these sequelae prolong the duration of mechanical ventilation and, consequently, the patient’s stay in the ICU. Therefore, developing effective secretion clearance strategies, including automated detection and appropriately timed secretion removal, is critical for mechanically ventilated patients.

The effectiveness of mechanical ventilation is primarily determined by the interplay of airway resistance, lung compliance and patient-ventilator synchrony, which together regulate ventilation dynamics, airflow distribution, and the driving pressure required for adequate gas exchange. Several studies have demonstrated a significant increase in airway resistance when secretions accumulate in the airways, particularly in the trachea and smaller bronchioles (Hess, 2014; Jubran and Tobin, 1994; Guglielminotti et al., 2000). This increased resistance necessitates higher ventilator pressures, including elevated peak inspiratory pressure (PIP), and increases the work of breathing to achieve adequate tidal volumes (Hess, 2014). In addition, positive end-expiratory pressure (PEEP) may require adjustment to prevent airway collapse and maintain alveolar recruitment, leading to higher inflation pressures that elevate the risk of ventilator-induced lung injury (VILI), as noted in Jubran and Tobin (1994); Guglielminotti et al. (2000); Zamanian and Marini (2006). When secretions obstruct the airways, gas exchange efficiency declines, resulting in a ventilation-perfusion mismatch as oxygenated air fails to reach certain lung regions due to mucus plugging. This can result in hypoxemia (low blood oxygen) and hypercapnia (elevated CO_2_), both of which are detrimental to critically-ill patients (Hess, 2007). Airway mucus also alters the lung tissue’s elastic properties, making it stiffer and less compliant, thereby necessitating higher ventilator pressures to maintain adequate oxygenation (Hess, 2014). Hence, early detection of secretion accumulation and its clearance through methods such as suctioning, humidification, and airway clearance techniques (e.g., respiratory physiotherapy) are essential for successful weaning (Goetz et al., 2022). Conventional indicators of secretion accumulation, such as coughing and audible respiratory sounds, are often unreliable in mechanically ventilated patients, particularly those who are sedated or have impaired cough reflexes. Consequently, clinicians turn to ventilator waveforms (VWFs), which are time series of pressure, flow, and volume signals, along with their loops, for secretion detection (Jubran and Tobin, 1994; Guglielminotti et al., 2000; Zamanian and Marini, 2006; Albani et al., 2021b; a; Harris, 2005). Visual interpretation of these waveforms remains challenging, however, due to factors like lung co-morbidities and patient-ventilator asynchronies (Younes et al., 2007; De Wit, 2011), which can obscure secretion-related changes (Paratz and Ntoumenopoulos, 2014). These issues underscore the need for non-invasive secretion quantification techniques based on bedside measurements to improve timely detection and management of airway secretions. To address this, we adopt a structured approach that begins by characterising the impact of secretion accumulation on respiratory dynamics and identifying corresponding signatures in ventilator waveforms. Our methodology is organized into three main stages:

• Model development and Validation: A computational model of pressure support ventilation is developed to simulate the effects of secretion accumulation on respiratory dynamics. The model incorporates non-linear airway resistance, lung compliance, and patient effort, and is validated against clinical ventilator waveform data.

• Feature Identification from Waveforms: The model is used to generate synthetic ventilator waveforms across varying secretion levels, enabling the extraction of key features that show consistent and quantifiable changes with secretion presence and clearance.

• Clinical Application and Evaluation: The extracted features are applied to patient ventilator data, and a secretion index is introduced as a composite metric for quantifying secretion accumulation and assessing its clinical relevance for non-invasive detection.

The remainder of this paper is organised as follows: Section 2 presents the development of a pressure support ventilator model incorporating non-linear resistance, compliance, and patient respiratory effort, along with details of its simulation, validation, and optimisation using clinical waveform data. Section 3 focuses on the application of this model for quantifying airway secretions, including analysis on both synthetic and real patient data, and introduces the secretion index as a novel metric for tracking secretion accumulation. Section 4 summarizes the key findings and discusses the translational potential of the proposed framework for real-time, non-invasive monitoring of secretion accumulation.

## 2 Mechanistic modelling for interpretability of ventilator waveforms

Mechanistic modelling of patient-ventilator dynamics plays a crucial role in understanding airway secretion clearance by capturing the interplay between airway resistance, lung compliance, and mode of ventilation. In addition, it provides information on VWF alterations associated with secretion accumulation. When calibrated with bedside data such as airway pressures, including peak inspiratory pressure (PIP) and plateau pressure (Pplat); tidal volume $\left(V_{\text{T}}\right)$ ; respiratory rate (RR); inspiratory and expiratory times; oxygenation and blood gas levels; patient-specific parameters such as height, weight, and lung pathology; secretion-related observations like suctioning frequency and secretion volume; respiratory effort metrics, including esophageal pressure (Pes); and ventilator settings such as, positive end-expiratory pressure (PEEP), and fraction of inspired oxygen (FiO_2_), the model can closely reflect patient-specific physiology and enhance its clinical applicability.

Early attempts at ventilator modelling relied primarily on simple equation-based approaches, such as linear resistance-compliance (RC) models, described in Bates (2009), which model the airflow using basic relationships between pressure, flow, and volume. In these models, the respiratory system is often represented by the equation

$$\Delta P_{aw}=R_{rs}\cdot Q_{aw}+V/C_{rs},$$ (1)

where $\Delta P_{aw}=P_{aw}-\text{PEEP}$ denotes airway pressure difference across the system, $Q_{aw}$ is the flow rate, V is the system volume obtained by integrating $Q_{aw}$ over time. $R_{rs}$ and $C_{rs}$ represents the airway resistance and total respiratory compliance respectively. This equation illustrates how changes in pressure are influenced by airway resistance and lung compliance, providing a foundational understanding of respiratory mechanics. While useful for approximating ventilator mechanics of sedated patients, these simple equations fail to capture the complex, non-linear, and time-dependent dynamics of patient-ventilator interactions (Chen et al., 2024). Overcoming these limitations requires more complex models and feedback mechanisms to dynamically model changes in airway resistance, compliance, and pressures, ensuring better alignment with clinical data (Albanese et al., 2016; Ngo et al., 2018). Compartmental models based on ordinary differential equations (ODEs) remain the most widely used approach, simplifying the complexity of the respiratory system by dividing it into compartments representing the lungs, airways, and surrounding anatomical structures. These models capture key dynamics through non-linear variations in parameters such as lung compliance and airway resistance (Liu et al., 1998; Sundaresan et al., 2009; Zhou et al., 2021). Due to their computational efficiency, they are widely employed to simulate basic ventilator modes, such as volume-controlled and pressure-controlled ventilation. More complex ventilator models build upon compartmental approaches by incorporating detailed anatomical and physiological features, including multi-compartment lung structures, complex airway branching with non-linear resistances, and dynamic interactions with the heart and surrounding blood vessels (Mistry et al., 2022; Cabeleira et al., 2024). While these models can replicate conditions such as acute respiratory distress syndrome (ARDS) and chronic obstructive pulmonary disease (COPD), they require patient-specific parameters and detailed representations of lung morphology to yield physiologically meaningful simulations.

Despite their utility, existing ventilator models struggle to accurately simulate advanced ventilator modes such as proportional assist ventilation (PAV) (Younes, 2002), continuous positive airway pressure with pressure support (CPAP-PS), and neurally adjusted ventilatory assist (NAVA), which depend on real-time patient feedback (Sinderby et al., 1999). Further, they often assume ideal synchronisation between the ventilator and the patient, overlooking asynchronies such as delayed triggering or breath stacking. Therefore, to better reflect real patient-ventilator interactions, it is essential to design models that incorporate dynamic feedback across various ventilator modes. Such physiology-informed models will also be crucial for enabling future advancements, including the integration of artificial intelligence for personalized ventilation, real-time secretion detection, patient effort monitoring, and prediction of ventilator asynchronies. These improvements necessitate models that continuously adapt to patient-ventilator interactions, enabling the simulation of diverse ventilator modes and accommodating a wide range of patient conditions.

In this study, we present a comprehensive ventilator model that incorporates airway resistance, lung compliance, chest wall mechanics, and patient-driven breath cycles to simulate pressure support ventilation. An event-based simulation strategy using callback functions enables real-time adjustment of ventilator pressure by tracking physiological transitions, such as peak inspiratory flow and its decline, allowing for realistic simulation of pressure support mode with dynamic feedback. Our model aims to uncover changes in VWFs in relation to underlying physiological parameters, particularly those influenced by secretion accumulation. Using the simulated waveforms, we extract key respiratory features such as peak inspiratory flow $\left(Q_{\text{max}}\right)$ , which represents the maximum airflow achieved during inspiration and reflects the combined effect of patient effort and airway resistance; tidal volume $\left(V_{\text{T}}\right)$ , the total volume of air delivered to the lungs during a single breath and an indicator of overall ventilation effectiveness; and the expiratory time constant (τ) , which characterizes how quickly the lungs empty during exhalation and is sensitive to both airway resistance and lung compliance. In a simplified single-compartment model, $\tau =R_{rs}C_{rs}$ , where $R_{rs}$ and $C_{rs}$ denote the airway resistance and respiratory system compliance, respectively. These features are then used to assess the potential of waveform-based analysis for real-time, non-invasive secretion monitoring and personalized respiratory care.

### 2.1 Model formulation

**FIGURE 1:**
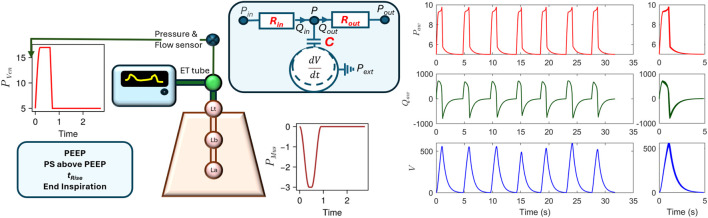
Left: The compartment model of pressure support mechanical ventilation. The model includes a feedback element that monitors inspiratory flow to guide the ventilator pressure profile. Right: A short recording of VWFs for 30 s and superimposition of individual breaths.

The mechanistic model developed in this study is designed to generate ventilator waveforms that reflect both clear and secretion-accumulated airways by modulating airway resistance, lung compliance, and patient effort parameters. Our model employs a compartmental modelling approach, where the mechanical ventilation system is represented as a series of interconnected compartments, each corresponding to specific components such as the ventilator, endotracheal tube, upper airways (trachea and bronchi), and smaller airways (alveoli). The bronchi and alveoli compartments are enclosed within a larger compartment referred to as the chest cavity that undergoes pressure changes due to the motion of the diaphragm. (See Figure 1). In this approach, each compartment is assigned parameters, such as resistance and compliance, to account for pressure changes and air storage capacity, respectively, as air moves in and out of the lungs.

A typical compartment is composed of a capacitor connected to inflow and outflow resistances to simulate the inflow and outflow of air from a compartment. The underlying governing equations used to compute the compartment’s internal pressure (P) , the flow in $\left(Q^{\text{in}}\right)$ , the flow out $\left(Q^{\text{out}}\right)$ and the volume change from some reference value $\left(V-V_{0}\right)$ are:

$$\frac{dV}{dt}=Q^{\text{in }}-Q^{\text{out }}.$$ (2)

$$Q^{\text{in }}=\frac{P^{\text{in }}-P}{R^{\text{in }}},\;Q^{\text{out }}=\frac{P-P^{\text{out }}}{R^{\text{out }}},$$ (3)

$$P=\frac{\left(V-V_{0}\right)}{C}+P_{\text{ext}},$$ (4)

These equations are formulated based upon principles of conservation laws, constitutive equations, and phenomenological relations. Equation 2 is the conservation equation that describes the dynamics of air inflow, outflow, and accumulation within the system, maintaining the integrity of volume conservation. The flow in and out of a compartment is then calculated using Equation 3, which depends on the pressure difference between connected compartments and the associated resistances. Equation 4 is the constitutive equation that computes the pressure in a compartment, with reference to an external pressure $P_{\text{ext}}$ , based on its compliance and the volume it occupies at that instant of time. The model consists of five interconnected compartments representing different regions of the respiratory system: the endotracheal (ET) tube, trachea (Lt), bronchi (Lb), alveoli (La), and thoracic (Thx) cavity. Each compartment exhibits specific compliance behaviour, denoted as $C_{\text{ET}},C_{\text{Lt}},C_{\text{Lb}},C_{\text{La}},C_{\text{Thx}}$ , which represent the elastic properties of the airway and lung structures. The airflow between these compartments is regulated by four resistances, $R_{\text{VenET}},R_{\text{ETLt}},R_{\text{LtLb}},R_{\text{LbLa}}$ , corresponding to different airway segments, from the ventilator to the alveoli. Additionally, each compartment has an associated unstressed volume $V_{0}$ , representing its equilibrium volume in the absence of external forces. The actual volume V at any given time deviates from this baseline, leading to a dynamic pressure evolution over time within the system. For the endotracheal tube and trachea compartments, the external pressure term, $P_{\text{ext}}$ , is set to $P_{\text{atm}}$ , which is the atmospheric pressure. Conversely, the thorax pressure $P_{\text{Thx}}$ is the external pressure for the bronchi and alveoli compartments within the chest cavity. The thoracic pressure, $P_{\text{Thx}}$ , is computed as:

$$P_{\text{Thx}}=\frac{V_{\text{Lb}}+V_{\text{La}}-V_{0\text{Thx}}}{C_{\text{Thx}}}+P_{\text{Mus}},$$ (5)

where $V_{\text{Lb}}$ and $V_{\text{La}}$ are the volumes of the bronchi and alveoli compartments, $C_{\text{Thx}}$ is the thoracic compliance, and $P_{\text{Mus}}$ is negative pressure generated by the respiratory muscles. Whilst the temporal dynamics of each compartment are described using ODEs, the spatial dependencies between different compartments are approximated through the implementation of lumped properties and the interconnections between the compartments. It is clear from the equations (Equations 1–5) that the compliance and resistances dictate the temporal evolution of the pressure and volume in each compartment. The compliances and resistances can be either constant or variable, with the potential for non-linear relationships to physiological variables depending on the specific compartment and its interactions with others, resulting in phenomenological equations that describe these dynamics. These phenomenological equations introduce non-linearity into the model by representing airway resistance and compliance as functions of flow and pressure, respectively, as proposed in Bates (2009). Specifically, the nonlinearity in airway resistance is modelled using the Rohrer’s equation (Flevari et al., 2011), which is applied to the two resistance segments between endotracheal tube to trachea and trachea to bronchi expressing the pressure-flow relationship as

$$R\left(Q\right)=K_{1}+K_{2}\cdot |Q|.$$ (6)

Here, $K_{1}$ and $K_{2}$ represent laminar and turbulent flow contributions to resistance. This introduces flow-dependent resistance, where resistance increases with higher airflow due to turbulent effects in the airway, making the ventilator mechanics highly non-linear. Specifically, in the context of secretion accumulation, we focus on modulating the laminar component $\left(K_{1}\right)$ of airway resistance, as mucus accumulation is assumed to primarily increase frictional resistance within the airways, particularly in the lower flow regimes typical of mechanically ventilated patients. The $K_{2}$ parameter, representing turbulent contributions, is kept constant, as the effects of turbulence are considered secondary under these conditions. While Rohrer’s equation captures flow-dependent resistance, this approach simplifies secretion effects to changes in resistance and does not explicitly account for mucus rheology. The lung compliance follows a modified version of Salazar-Knowles sigmoidal model (Salazar and Knowles, 1964), given by

$$C_{La}=C_{\text{base}}\left(1-\alpha e^{-\beta P_{La}}\right),$$ (7)

where compliance varies dynamically with alveolar pressure $P_{La}$ , capturing the gradual stiffening of the lungs at higher pressures. The parameters α and β in the model regulate the compliance adaptation. In this case, α determines the magnitude of compliance reduction, while β controls the rate at which compliance decreases as $P_{La}$ increases, effectively capturing the progressive stiffening of lung tissue at higher pressures (Cabeleira et al., 2024). This non-linear compliance response ensures the model accurately represents pressure-volume dynamics observed in real lungs, particularly during mechanical ventilation (Liu et al., 1998; Polak and Mroczka, 2006). The non-linear resistances and compliances as described in Equations 6, 7 are specific to the patient or scenario and need to be fitted accordingly for accurate physiological representation. In a typical pressure support mode, the ventilator pressure $\left(P_{\text{Ven}}\right)$ serves as the driving pressure, controlling airflow into the lung compartments by cycling between inspiratory and expiratory pressures. This pressure is generated according to the ventilator’s settings, including PEEP, PS above PEEP, and the timing parameters of the breathing cycle, creating a waveform that induces mechanical ventilation. The ventilator pressure waveform $P_{\text{Ven}}\left(t\right)$ is assumed here to take the following form:

$$P_{\text{Ven}}\left(t\right)=\left\{\begin{array}{cc} \text{PEEP}+P_{\text{Insp}}^{\text{Ven}}\left(t\right),\; & \text{if }0\leq t<t_{\text{RiseVT}}\\ \text{PS above PEEP},\; & \text{if }t_{\text{RiseVT}}\leq t<t_{\text{InspVT}}\\ \text{PS above PEEP}-P_{\text{Exp}}^{\text{Ven}}\left(t\right),\; & \text{if }t_{\text{InspVT}}\leq t<t_{\text{InspVT}}+t_{\text{Valve}}\\ \text{PEEP},\; & \text{otherwise,} \end{array}\right.$$ (8)

where the time variable t is measured relative to the start of each ventilator cycle of duration $t_{\text{cycleVT}}$ (i.e., $t\in \left[0,t_{\text{cycleVT}}\right)$ within each cycle), PEEP is the positive end-expiratory pressure, PS above PEEP is the pressure support above PEEP, $t_{\text{RiseVT}}$ is the time taken to reach peak pressure, $t_{\text{InspVT}}$ is the duration of inspiration, and $t_{\text{Valve}}$ is the valve closing time during the transition to expiration. Specifically, the distinct shape of this pressure waveform is given here by the functions:

$$\begin{array}{ll} P_{\text{Insp}}^{\text{Ven}}\left(t\right) & =\left(\text{PS above PEEP}-\text{PEEP}\right)\sin\left(\frac{\pi \cdot t}{2\cdot t_{\text{RiseVT}}}\right)\text{and}\\ P_{\text{Exp}}^{\text{Ven}}\left(t\right) & =\left(\text{PS above PEEP}-\text{PEEP}\right)\frac{t-t_{\text{InspVT}}}{t_{\text{Valve}}}. \end{array}$$

The respiratory muscle pressure $P_{\text{Mus}}\left(t\right)$ is modeled using a piecewise function to simulate the inspiratory and expiratory phases of spontaneous breathing. The pressure waveform is governed by the patient’s respiratory effort and defined in terms of baseline and peak effort parameters:

$$P_{\text{Mus}}\left(t\right)=\left\{\begin{array}{cc} P_{\text{base}}+\frac{1}{2}\left(P_{\text{peak}}-P_{\text{base}}\right)\left[1-\cos\left(\frac{\pi t}{t_{\text{RisePT}}}\right)\right],\; & \text{if }0\leq t<t_{\text{RisePT}}\\ P_{\text{peak}},\; & \text{if }t_{\text{RisePT}}\leq t<t_{\text{InspPT}}\\ P_{\text{base}}+\frac{1}{2}\left(P_{\text{peak}}-P_{\text{base}}\right)\left[1+\cos\left(\frac{\pi \left(t-t_{\text{InspPT}}\right)}{t_{\text{ExpPT}}}\right)\right],\; & \text{if }t_{\text{InspPT}}\leq t<t_{\text{InspPT}}+t_{\text{ExpPT}}\\ P_{\text{base}},\; & \text{otherwise} \end{array}\right.$$ (9)

This formulation simulates a smooth pressure profile for a spontaneous breath. During the inspiratory rise phase $\left(0\leq t<t_{\text{RisePT}}\right)$ , the muscle pressure decreases from the baseline value $P_{\text{base}}$ to a more negative peak $P_{\text{peak}}$ , reflecting the onset of active inspiratory effort. This is followed by a sustained inspiratory phase $\left(t_{\text{RisePT}}\leq t<t_{\text{InspPT}}\right)$ where pressure remains at $P_{\text{peak}}$ . During the early expiration phase $\left(t_{\text{InspPT}}\leq t<t_{\text{InspPT}}+t_{\text{ExpPT}}\right)$ , the pressure gradually returns to baseline, modelling passive muscle relaxation. For the remainder of the respiratory cycle, the pressure stays constant at $P_{\text{base}}$ until the onset of the next breath. This expression allows for physiologically realistic transitions and control of breath phase timing and effort. In order to incorporate patient–ventilator interaction nature of CPAP-PS mode, the model includes a feedback component (described in the next section) that detects key phases of spontaneous breathing, such as peak inspiratory flow and end-inspiratory timing. This feedback informs the timing and duration of pressure support, allowing for optimal airflow delivery into the lungs. Although this study focuses on the CPAP-PS ventilation mode, the model architecture is readily adaptable to other commonly used modes. By modifying the ventilator pressure waveform $P_{\text{Ven}}\left(t\right)$ in Equation 8, the model can simulate pressure control ventilation (PCV) or mandatory breaths in synchronized intermittent mandatory ventilation (SIMV). In such cases, the patient feedback component can be disabled, and $P_{\text{Ven}}\left(t\right)$ can be predefined as a square waveform, where the PS above PEEP functions as the peak inspiratory pressure (PIP) applied over a fixed inspiratory period. This flexibility supports potential extensions of the framework for broader ICU scenarios, including controlled ventilation phases or spontaneous breathing trials.

### 2.2 Model simulation including feedback

Forward simulations were performed to generate VWFs for CPAP-PS ventilation. In this mode, the ventilator provides pressure support (PS) above positive end-expiratory pressure (PEEP) until the inspiratory flow in the airway decreases to a predefined threshold, typically set at 20% or 30% of the peak inspiratory flow rate. The simulation incorporates a predefined patient spontaneous breathing profile, $P_{\text{Mus}}\left(t\right)$ , along with ventilator settings such as rise time, PEEP, PS above PEEP, and end-inspiration flow timing to generate a feedback-dependent ventilator pressure, $P_{\text{Ven}}\left(t\right)$ . Table 1 provides an overview of the model input parameters used to configure the simulation and the resulting outputs, including ventilator waveforms and derived quantities. The model parameters such as resistances and compliances were based on data from the literature, specifically Cabeleira et al. (2024), and listed in Table 2. In CPAP-PS mode, the ventilator assists patient-initiated breaths by maintaining a continuous positive airway pressure throughout the respiratory cycle while providing additional pressure during inspiration to support the patient’s spontaneous breathing effort. To realistically simulate the ventilator dynamics, we use an event-based simulation approach, which is well-suited for modelling CPAP-PS, by providing feedback to dynamically control subsequent ventilator pressures in response to patient respiratory muscle pressures generated by $P_{\text{Mus}}\left(t\right)$ using Equation 9. The function $P_{\text{Ven}}\left(t\right)$ represents both the timing and magnitude of CPAP-PS support, dynamically adjusting ventilator pressures based on patient effort and using event-based triggers to capture critical transition points.

**TABLE 1:** Summary of model input parameters and output.

Input parameters	
PEEP, PS above PEEP	Ventilator settings: Positive End-Expiratory Pressure and Pressure Support above PEEP
$R_{\text{VenET}}$ , $R_{\text{LbLa}}$ , $K_{1}^{\text{ETLt}},K_{1}^{\text{LtLb}}$ , $K_{2}^{\text{ETLt}},K_{2}^{\text{LtLb}}$	Airway resistance parameters, including laminar $\left(K_{1}\right)$ and turbulent $\left(K_{2}\right)$ flow resistance components
$C_{\text{ET}},C_{\text{Lt}},C_{\text{Lb}},C_{\text{base}},C_{\text{Thx}}$ , α,β	Compliance parameters of compartments and compliance shaping terms from the Salazar-Knowles model
$P_{\text{peak}},t_{\text{RisePT}},t_{\text{InspPT}},t_{\text{ExpPT}}$	Patient respiratory muscle pressure parameters
$t_{\text{cycleVT}},t_{\text{RiseVT}},t_{\text{Valve}}$	Ventilator cycle timing parameters
End Inspiration %	End-inspiration flow threshold: defines the percentage of peak inspiratory flow at which the ventilator switches from inspiration to expiration

Model Outputs	
$P_{\text{aw}}\left(t\right)$ , $Q_{\text{aw}}\left(t\right)$ , V(t)	Ventilator waveforms: airway pressure, airway flow, and lung volume
$Q_{\text{max}}$ , $V_{T}$ , τ	Extracted features: peak inspiratory flow, tidal volume, and expiratory time constant

**TABLE 2:** Model parameters and their ranges used for simulating CPAP-PS mechanical ventilation. *Indicates parameters included in the 14-dimensional optimization vector.

Parameter	Value/Range
**Cycle Timing Parameters**	
$t_{\text{cycle,PT}}$	2.0–3.0 s
$t_{\text{cycle,VT}}$	2.0–3.0 s
$t_{\text{RisePT}}^{*}$	0.15–0.25 s
$t_{\text{InspPT}}^{*}$	0.75–1.00 s
$t_{\text{ExpPT}}^{*}$	0.3–0.5 s
**Pressure Parameters**	
$P_{\text{peak}}^{*}$	−5.0 to −1.0 mmHg
PEEP	5.0 mmHg
PS above PEEP	10.0 mmHg
**Lung Compliance Parameters**	
$C_{\text{base}}^{*}$	10.0–100.0 mL/mmHg
$C_{\text{Lb}}^{*}$	1.0–10.0 mL/mmHg
$C_{\text{Lt}}^{*}$	1.0–10.0 mL/mmHg
$C_{\text{ET}}^{*}$	1.0–10.0 mL/mmHg
$C_{\text{Thx}}^{*}$	300.0–500.0 mL/mmHg
α	0.05
$\beta^{*}$	0.03–0.05 ${\text{mmHg}}^{-1}$
**Airway Resistance Parameters**	
$R_{\text{VenET}}^{*}$	0.0005–0.005 mmHg ⋅ s/mL
$K_{1,\text{ETLt}}^{*}$	0.0005–0.005 mmHg ⋅ s/mL
$K_{1,\text{LtLb}}^{*}$	0.0005–0.005 mmHg ⋅ s/mL
$K_{2,\text{ETLt}}$	$1\times 10^{-6}$ mmHg $\cdot {\text{s}}^{2}$ / ${ml}^{2}$
$K_{2,\text{LtLb}}$	$1\times 10^{-6}$ mmHg $\cdot {\text{s}}^{2}$ / ${ml}^{2}$
$R_{\text{LbLa}}^{*}$	0.0005–0.005 mmHg ⋅ s/mL
**Lung Volume Parameters**	
$V_{0\text{La}}$	2000.0 mL
$V_{0\text{Lb}}$	70.0 mL
$V_{0\text{Lt}}$	70.0 mL
$V_{0\text{ET}}$	70.0 mL
$V_{0\text{Thx}}$	3,500.0 mL

The governing ODEs described in the model formulation section are implemented and solved using the Julia programming language, leveraging the efficiency and flexibility of Julia’s scientific computing ecosystem. The DifferentialEquations.jl package is used for numerical integration of the governing equations, ensuring robust time-dependent simulations (Rackauckas and Nie, 2017). Additionally, as in Pal et al. (2024), NLsolve.jl and Roots. jl are employed to solve the non-linear equations for resistance and compliance, allowing for a physiologically accurate representation of airway dynamics. We employ a fourth-order Runge–Kutta (RK4) method for time integration of the ventilator model, supplemented with event-driven callbacks to detect specific physiological transitions during the respiratory cycle. These callbacks are used to identify key features such as the peak inspiratory flow rate $\left(Q_{\text{max}}\right)$ and to track when the flow decays to a defined fraction of this peak, capturing critical phase transitions in the ventilator waveform. In our model, the variables $P_{\text{ET}}$ and $Q_{\text{VenET}}$ correspond to the airway pressure $\left(P_{aw}\right)$ and airway flow $\left(Q_{aw}\right)$ , respectively. The first callback detects the maximum flow rate, identifying the time point near $t_{\text{RiseVT}}$ that marks the rise in ventilator pressure. Upon reaching this peak flow, the simulation records $Q_{\text{max}}$ and continues with the computation of subsequent phases. The second callback identifies when flow in the airway declines to 20% or 30% of its peak, signalling the end of inspiration and transition to expiration. This event-based approach ensures that each phase of the ventilator cycle is aligned with the physiological timing, accurately reflecting the interaction between the ventilator-applied pressure and the patient’s respiratory mechanics. In particular, the $P_{\text{Ven}}\left(t\right)$ and $P_{\text{Mus}}\left(t\right)$ together drive the dynamic response of the respiratory system in the model, influencing the compartmental pressures and airflow rates, thus providing insights into the combined effect of mechanically- and physiologically-induced pressure changes on the airway pressure distribution and ventilation dynamics.

### 2.3 Model validation and optimisation

To validate the CPAP-PS ventilator model, clinical VWF data was used to compare simulated and recorded patient waveforms. VWF data was obtained from the Getinge Servo-U ventilator (Göteborg, Sweden), which provides real-time monitoring and recording of flow, volume, and pressure waveforms. During routine airway clearance procedures, pre-suction waveform data was recorded using the ventilator’s built-in data acquisition system before performing an airway clearance procedure to remove excess secretions. The post suction waveform data was obtained at least 2 minutes post procedure to allow for resumption of the baseline respiratory rate under identical ventilator settings to ensure comparability. Both pre- and post-suction waveform datasets were exported and the data parsed and stored in a structured, human-readable format for further analysis. This structured data acquisition process ensured consistency in ventilator measurements, enabling reliable comparison of waveform changes associated with secretion removal. The secretion levels were assessed by an expert physiotherapist using standard clinical criteria during suctioning. These were initially recorded as five categories: none, small, moderate, large, and copious. For the purposes of analysis, these were aggregated into three broader categories by grouping none and small as low, moderate as medium, and large and copious as high secretion levels. Unfortunately, direct quantitative secretion volume measurements were not routinely available for all patients, but the categorization based on clinical judgement was consistent with routine ICU practice.

**FIGURE 2:**
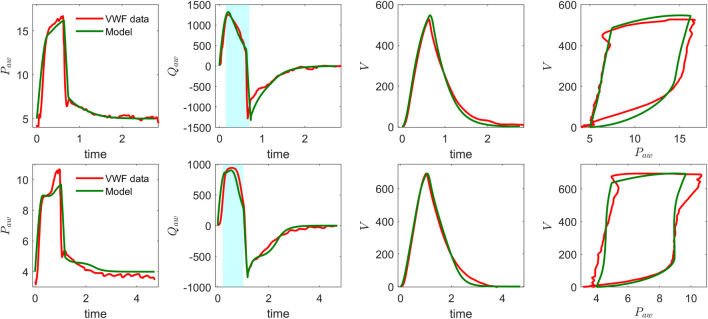
Model validation of CPAP-PS simulation using real ventilator waveforms: airway pressure, airway flow, volume, and pressure–volume loop for two patients. Note the differences in airway pressure and flow patterns between the top and bottom plots, particularly during the inspiratory phase (highlighted in the shaded region of the flow curves).

**FIGURE 3:**
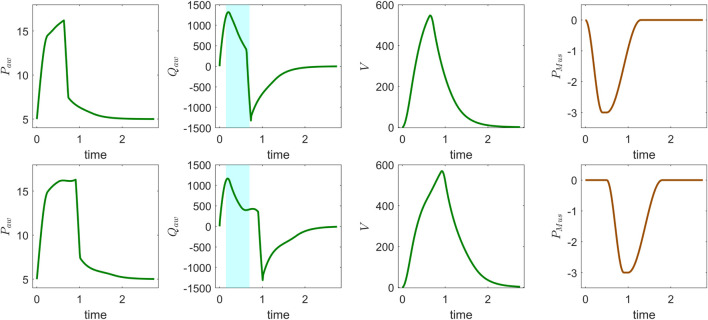
Model simulation of CPAP-PS mode using patient-specific parameters: airway pressure $\left(P_{aw}\right)$ , airway flow $\left(Q_{aw}\right)$ , lung volume (V) , and muscle pressure $\left(P_{\text{Mus}}\right)$ for two different patient effort scenarios. Note the differences in airway pressure and flow profiles between the simulations, especially during the inspiratory phase (highlighted in the shaded region), which reflect variations in spontaneous breathing effort and patient–ventilator interaction.

The ventilator model simulations were compared with clinical ventilator waveform data by running forward simulations and manually adjusting the model parameters to improve alignment with observed pressure, flow, and volume waveforms over time. Figure 2 presents ventilator waveforms from clinical cases, demonstrating the model’s ability to capture physiological changes and provide meaningful interpretations of patient-specific ventilator dynamics. In Figure 2 (top), a linear decrease in the flow curve is observed during the interval between peak flow and the end of inspiration, indicating a passive decay of inspiratory flow as pressure support is maintained constant. In contrast, Figure 2 (bottom) shows a sharp increase near the latter part of inspiration, suggesting that the patient’s breathing effort is dominating ventilator support. This is further evident in the concave shape of the flow curve during the transition from rise time to end of inspiration. To further illustrate the clinical relevance of the model, we present another example that reflects distinct patient–ventilator interaction patterns observed in clinical data. Figure 3 shows model-based simulations for two different patient effort scenarios. In the first case (top panel), the patient initiates a spontaneous breath which is promptly supported by the ventilator, demonstrating synchrony between patient effort and ventilator support. In the second case (bottom panel), a spontaneous breath is initiated by the patient before the mandatory ventilator breath could complete, resulting in patient–ventilator desynchrony and double triggering, which is clearly evident in the flow and pressure waveforms. The model demonstrates a strong capability in capturing patient-ventilator dynamics, effectively reflecting changes in airway resistance, compliance, and patient effort within ventilator waveforms. Despite its minimal formulation, which simplifies the spatial structure of the lung by representing key anatomical regions, such as the trachea, bronchi, and alveoli, using lumped resistance $\left(R_{\text{LtLb}},R_{\text{LbLa}}\right)$ and compliance $\left(C_{\text{Lt}},C_{\text{Lb}},C_{\text{La}}\right)$ parameters, the model maintains spatial simplicity while providing sufficient temporal accuracy to capture subtle VWF variations arising from patient–ventilator interactions, all with minimal computational complexity. The choice of model parameters and operating range helps to ensure that the solutions accurately reflect the VWFs. In practical implementations, incorporating physiological constraints, such as those based on expected muscle pressure dynamics, into the parameter identification process can ensure that the model adheres to realistic physiological limits (Vicario et al., 2015).

In our study, we calibrated the ventilator model and estimated optimal parameter values using an evolutionary optimization approach. Specifically, we employed a non-gradient-based optimization method, Exponential Natural Evolution Strategy (xNES), developed initially by Glasmachers et al. (2010) and implemented using the BlackBoxOptim package in Julia. xNES is a derivative-free evolutionary optimization method designed for high-dimensional, non-linear objective functions. Its suitability for physiological model calibration arises from its ability to efficiently explore complex, noisy parameter spaces where solutions are often non-unique and interdependent. In ventilator simulations, where patient-specific variations introduce significant heterogeneity in the resulting lung mechanics, xNES enables robust parameter estimation without relying on gradient information, making it more effective than traditional optimization methods. The xNES optimization minimizes the discrepancy between simulated and observed patient data (pressure, flow, and volume waveforms) by refining model parameters within physiological constraints. It iteratively explores the parameter space, adapting its search distribution and covariance matrix to converge on an optimal solution that minimizes the fitness score. The process terminates when a target fitness or iteration limit is reached, yielding a calibrated parameter set that closely aligns the ventilator model with patient data. Mathematically, this involves minimizing the difference between simulated data $X_{\text{sim}}\left(t\right)$ and observed patient data $X_{\text{obs}}\left(t\right)$ across ventilator signals such as pressure $P_{aw}\left(t\right)$ , flow $Q_{aw}\left(t\right)$ , and volume V(t) . Dynamic Time Warping (DTW), a measure that accounts for both the amplitude and temporal alignment, is used to evaluate the fit. The objective function is defined as:

$$f\left(\theta \right)=\underset{i\in \left\{P,Q,V\right\}}{\sum }w_{i}\;\text{DTW}\left(X_{\text{sim},i}\left(\theta \right),X_{\text{obs},i}\right),$$

where θ represents the model parameters to be optimized, $X_{\text{sim},i}\left(\theta \right)$ is the simulated time-series data for variable i (e.g., pressure, flow, volume) given parameters θ , $X_{\text{obs},i}$ is the observed time-series data for variable i , $\text{DTW}\left(X_{\text{sim},i}\left(\theta \right),X_{\text{obs},i}\right)$ computes the DTW distance between the simulated and observed data for variable i , and $w_{i}$ is a weighting factor for each variable i . The DTW measures the similarity between two time-series, particularly focusing on the temporal alignment of the maxima and minima. This approach enables the optimization to achieve a close fit between the simulated and observed ventilator waveforms. xNES is used to explore the parameter space by adjusting the search distribution, where parameter samples θ are drawn from a multivariate normal distribution θ∼N(μ,C) , where μ is the mean vector of the parameter distribution, and C is the covariance matrix. At each iteration, xNES updates μ and C based on sampled parameters θ and their fitness scores. More details on the method can be found in Glasmachers et al. (2010). The optimization terminates when either the fitness score f(θ) meets a predefined threshold $f_{\text{target}}$ or the iteration count t exceeds the maximum limit $T_{\max}$ , *i.e.,*

$$\text{Stop if }f\left(\theta \right)\leq f_{\text{target}}\text{ or }t\geq T_{\max}.$$

**FIGURE 4:**
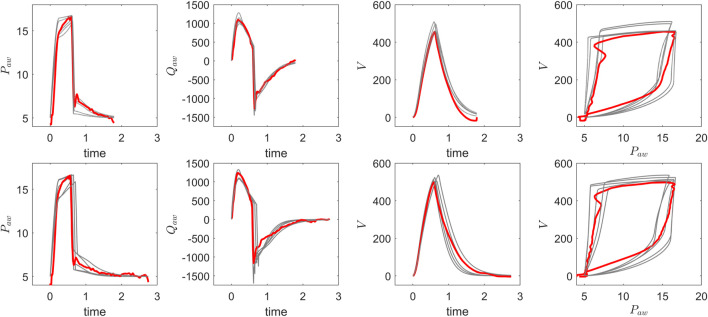
Comparison of optimisation results for pressure, flow, and volume waveforms: pre-suction (top) and post-suction (bottom). The recorded VWF data (red) is overlaid with five optimized VWF profiles (grey), demonstrating consistent alignment between the recorded data and optimised solutions before and after secretion clearance.

The optimization is performed over a 14-dimensional parameter vector, denoted as θ , consisting of compliance, resistance, and spontaneous breathing parameters within the model. Specifically, the compliance components include $C_{\text{ET}},C_{\text{Lt}},C_{\text{Lb}},C_{\mathrm{base}},\beta ,C_{\text{Thx}}$ , representing the elastic properties of different respiratory compartments. The four resistance parameters, $R_{\text{VenET}},K_{1\text{ETLt}},K_{1\text{LtLb}},R_{\text{LbLa}}$ , that define airflow resistance across various airway segments. Finally, four spontaneous breathing parameters, $P_{\text{peak}},t_{\text{RisePT}},t_{\text{InspPT}},t_{\text{ExpPT}}$ , characterize the peak inspiratory effort, and the timing of different phases in the breathing cycle. The list of optimized parameters and their physiological bounds is provided in Table 2; these bounds serve as physiologically informed guidelines and can be adjusted to achieve the best fit for individual breaths. These parameters collectively form the search space explored by xNES to minimize the discrepancy between simulated and observed ventilator waveforms. To analyse the effect of suctioning on the VWFs, we optimized individual breaths before and after the suctioning procedure using the xNES algorithm. Due to the stochastic nature of xNES, we performed 10 optimization runs per breath to capture the range of possible solutions. This approach accounts for variability in the optimization process and enables a robust statistical comparison of parameter distributions before and after secretion removal by evaluating multiple plausible solutions per breath. Figure 4 compares pre-suction (top) and post-suction (bottom) ventilator waveforms for pressure, flow, and volume, illustrating that the optimization process generates multiple plausible parameter sets per breath, each capable of closely reproducing the observed VWFs. This non-uniqueness reflects a fundamental challenge in physiological modelling, as parameters related to respiratory muscle pressure cannot be uniquely estimated from ventilator waveforms alone. Addressing this limitation may require integrating oesophageal pressure measurements or applying stronger priors and Bayesian techniques to constrain the parameter space within physiologically plausible bounds (Tsaneva-Atanasova et al., 2025; Cheng et al., 2025). While formal confidence intervals were not calculated in this study, parameter ambiguity was mitigated by imposing physiological bounds during the xNES optimization (Table 2). To evaluate the model fit, we computed the Root Mean Square Error (RMSE) and the coefficient of determination $\left(R^{2}\right)$ for airway pressure $\left(P_{\text{aw}}\right)$ , flow $\left(Q_{\text{aw}}\right)$ , and volume (V) waveforms across the analysed breaths. The RMSE and $R^{2}$ values in Table A1 indicate good agreement between the model and experimental data across both pre- and post-suction breaths. RMSE values for airway pressure remained below 2.2 mmHg, and flow and volume errors were within physiologically reasonable ranges. The $R^{2}$ values were consistently above 0.75 for all signals, with most exceeding 0.85, confirming that the model accurately captures key features of patient-specific respiratory dynamics.

## 3 Secretion quantification using ventilator waveforms

The ability to detect and quantify airway secretion accumulation remains a critical yet unresolved challenge in mechanical ventilation. Traditional methods for assessing secretion accumulation have notable limitations. One common approach is auscultation, which involves listening to lung sounds with a stethoscope. This method is inherently subjective and often unreliable in sedated or intubated patients. Another common practice is secretion volume estimation, typically performed after suctioning, which provides only retrospective insight and relies on visual assessment and clinical judgment rather than standardized or predictive metrics. Although ventilator waveforms are routinely monitored at the bedside, their potential for secretion monitoring remains underutilized due to the absence of structured methods for quantifying secretion load and its impact on respiratory dynamics. To address this gap, we use our CPAP-PS model to simulate secretion-related waveform alterations, providing a controlled framework for understanding the impact of secretion accumulation on ventilation patterns and enabling the development of quantitative metrics for secretion detection. To quantify secretion accumulation, various features derived from ventilator waveform data were considered, including peak inspiratory flow $\left(Q_{\text{max}}\right)$ , tidal volume $\left(V_{\text{T}}\right)$ , and the expiratory time constant (τ) . The peak inspiratory flow was computed as the maximum value of the flow waveform during the inspiratory phase. Tidal volume was obtained from the peak of the volume waveform, corresponding to the maximum lung inflation in a breath cycle. The expiratory time constant (τ) was estimated by fitting an exponential decay model to the expiratory portion of the volume waveform and extracting the decay constant from the fitted curve. These waveform features capture both inspiratory and expiratory airflow characteristics, thus providing a basis for assessing secretion-induced changes in respiratory dynamics.

### 3.1 Secretion analysis on VWF synthetic data

**FIGURE 5:**
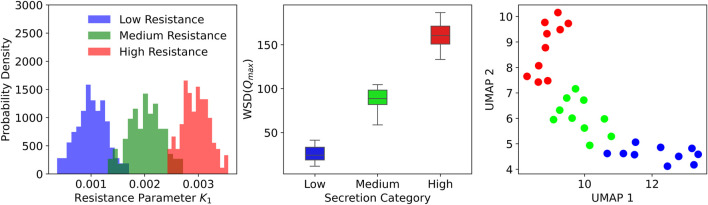
Distribution of airway resistance parameters and clustering based on ventilator waveform features. Left: Histograms of resistance values across three secretion levels. Middle: Box plots showing separation in WSD $\left(Q_{\max}\right)$ across secretion levels. Right: UMAP projection of patient clusters, with Low (blue), Medium (green), and High (red) secretion levels.

To evaluate the impact of secretion accumulation on ventilator waveforms, we generated a synthetic dataset using the CPAP-PS ventilator model developed in this study. Secretion levels were assumed here to be proportional to increased airway resistance, with specific parameter variations introduced to simulate different secretion accumulations. In this exploratory analysis, we selected resistance distributions to systematically probe how varying resistance levels affect ventilator waveforms. We defined a baseline resistance mean of 0.001 mmHg ⋅ s/mL with a standard deviation of 0.0003 mmHg ⋅ s/mL to capture variability in breaths. To examine moderate and high secretion scenarios, we shifted the mean to two- and threefold the baseline, respectively. This pragmatic approach allows us to identify waveform features that are indicative of changes in airway resistance. While this analysis focuses solely on airway resistance, confounding factors such as lung compliance and patient effort also influence these waveform features. In the model, the airway resistance parameters $K_{1\text{ETLt}}$ and $K_{1\text{LtLb}}$ were assumed to follow a normal distribution, with mean values of N(0.001,0.0003) for low resistance, N(0.002,0.0003) for medium resistance, and N(0.003,0.0003) for high resistance. Figure 5 (left) shows the typical airway resistance distributions corresponding to different secretion levels. The resistance increase was chosen to be twice and thrice the baseline value to represent medium and high secretion levels, respectively. To introduce breath-to-breath variability, the muscle pressure signal was varied for each simulated breath by randomly sampling parameters of $P_{\mathrm{Mus}}\left(t\right)$ , including $P_{\text{peak}}$ , $t_{\text{RisePT}}$ , $t_{\text{ExpPT}}$ , and the total cycle duration $\left(t_{\text{cyclePT}}\right)$ . For each simulated breath, the muscle pressure signal $\left(P_{\mathrm{Mus}}\right)$ was generated by sampling key parameters from uniform distributions: the total cycle time $t_{\text{cycle}}$ was drawn from U(4.0,5.0) , peak inspiratory pressure $P_{\text{peak}}$ from U(−5.0,−4.0) , inspiratory rise time $t_{\text{RisePT}}$ from U(0.5,1.0) , and expiratory time $t_{\text{ExpPT}}$ from U(0.1,1.0) . A total of 30 synthetic patients were simulated, equally distributed across three secretion levels (10 per category). Each patient underwent 12 simulated breaths pre-suction and 12 post-suction, resulting in 24 breaths per patient. Patients were categorized based on their pre- and post-suction resistance distributions. High secretion patients had a pre-suction resistance distribution of N(0.003,0.0003) and post-suction N(0.001,0.0003) , medium secretion patients had a pre-suction resistance of N(0.002,0.0003) and post-suction N(0.001,0.0003) , while low secretion patients maintained a resistance distribution of N(0.001,0.0003) before and after the suctioning procedure. The synthetic ventilator waveform data was then used to analyse the effects of secretion accumulation and removal on the ventilator waveforms.

A quantitative analysis was conducted to evaluate secretion-related changes in ventilator waveforms using the synthetic dataset. For each of the 30 synthetic patients, peak inspiratory flow $\left(Q_{\text{max}}\right)$ , tidal volume $\left(V_{\text{T}}\right)$ , and the expiratory time constant (τ) were computed over 12 simulated breaths before and after suctioning. The Wasserstein distance (WSD) was used to quantify the change in distribution of each feature across the pre- and post-suction states, resulting in three WSD scores per patient. Figure 5 (middle) shows a clear separation in the distributions of the peak inspiratory flows with different secretion levels. These WSD-derived metrics were then used as input to a Uniform Manifold Approximation and Projection (UMAP) algorithm, which clustered patients based on secretion accumulation. UMAP is a non-linear dimensionality reduction technique that preserves both local and global data structure, making it well-suited for clustering tasks in high-dimensional datasets (McInnes et al., 2018). It is particularly effective for visualizing complex data by projecting it into lower dimensions (e.g., 2D or 3D) while maintaining meaningful relationships between observations (patients, in this case). In this analysis, UMAP was applied as an unsupervised clustering method, and the resulting embeddings revealed distinct patient clusters corresponding to low, medium, and high secretion levels. These results suggest that waveform-derived features can provide sufficient information to categorize patients by secretion accumulation, highlighting the potential of ventilator waveform analysis as a non-invasive tool for secretion assessment.

### 3.2 Secretion analysis on VWF patient data

To extend the insights gained from synthetic VWF data analysis, we applied the same feature extraction methodology to real patient VWF data, where secretion levels were clinically assessed. The real-world VWF signals, recorded over short time intervals before and after airway clearance procedures, were inherently more variable and susceptible to noise. We analysed data from 35 patients, of whom four had copious secretions, seven had large, nine had moderate, 13 had small, and two had no detectable secretions. The analysis of individual breaths revealed noticeable shifts in the distributions of peak inspiratory flow rate following suctioning. However, changes in tidal volume and expiratory time constant were less discernible, likely due to signal variability and the subtle nature of secretion-related effects on these features. To provide a more detailed representation of the inspiratory phase, we introduced two slope-based metrics, namely, the flow slope and the volume slope. The flow slope $\left(Q_{\text{slope}}\right)$ quantifies the rate of decline in inspiratory flow from the point of maximum flow to the point of maximum volume, computed as $\left(Q_{\text{Qmax}}-Q_{\text{Vmax}}\right)/\left(t_{\text{Vmax}}-t_{\text{Qmax}}\right)$ . The volume slope $\left(V_{\text{slope}}\right)$ represents the rate of volume increase over this same interval, defined as $\left(V_{\text{Vmax}}-V_{\text{Qmax}}\right)/\left(t_{\text{Vmax}}-t_{\text{Qmax}}\right)$ . Here, $Q_{\text{Qmax}}$ denotes the peak inspiratory flow, while $V_{\text{Qmax}}$ is the lung volume at the time of this peak. Conversely, $V_{\text{Vmax}}$ corresponds to the maximum lung volume, and $Q_{\text{Vmax}}$ is the flow at that time. The interval between $t_{\text{Qmax}}$ and $t_{\text{Vmax}}$ captures the late-inspiratory segment, during which flow rate typically falls as the lungs approach peak inflation. This segment is particularly sensitive to airway resistance, lung compliance, and patient–ventilator interaction. The slope-based metrics introduced here characterize the temporal evolution of the inspiratory waveform, offering a quantitative description of breath mechanics. A reduced $Q_{\text{slope}}$ may indicate increased airway resistance or partial obstruction, potentially due to secretion accumulation. In contrast, deviations in $V_{\text{slope}}$ may be associated with changes in lung compliance or altered volume recruitment during inspiration. Together, these measures serve as physiologically meaningful indicators that support the detection and interpretation of secretion-related changes in the VWFs. To quantify the relationship between secretion levels and ventilator waveform features, we computed WSDs using $Q_{\text{max}}$ , $Q_{\text{slope}}$ , and $V_{\text{slope}}$ , thereby integrating both conventional and slope-based descriptors of VWFs.

**FIGURE 6:**
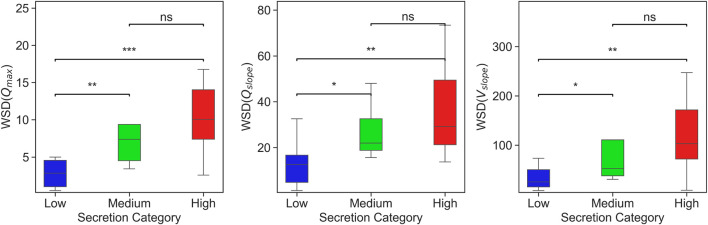
Box plots showing WSD-based considering three features ( $Q_{\max}$ , $Q_{\mathrm{slope}}$ , and $V_{\mathrm{slope}}$ ) across secretion categories: Low, Medium, and High. Asterisks indicate statistically significant differences between groups based on the Mann–Whitney U test: ^*^ p<0.05 , ^**^ p<0.01 , ^***^ p<0.001 ; *ns* denotes not significant.

**FIGURE 7:**
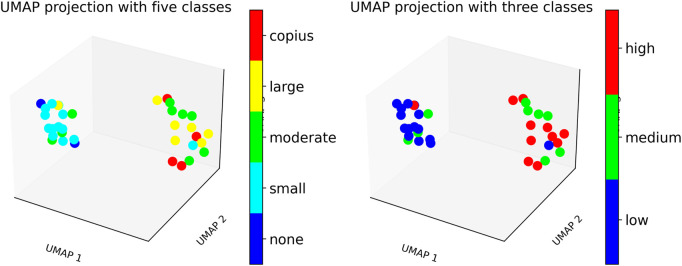
UMAP plot showing patient clustering based on secretion levels using WSD-based measures. Left: Patients categorized into five clinical secretion levels: none, small, moderate, large, and copious. Right: Patients grouped into three aggregated secretion levels: low, medium, and high.

Figure 6 presents a box plots comparing WSD scores across different secretion levels, showing that WSD derived from peak inspiratory flow rates effectively distinguishes between varying levels of secretion. Features such as $Q_{\text{slope}}$ , and $V_{\text{slope}}$ across different secretion categories reveals meaningful trends. Patients in the high secretion group exhibit significantly higher WSD values compared to those in the low group, indicating greater variability in their VWFs. This suggests that it is feasible to distinguish between low and high secretion accumulations using WSD-based metrics. However, the overlap in WSD values between the medium and high groups, as evidenced by non-significant statistical differences in several features, highlights the difficulty in clearly separating these two categories. These findings imply that while WSD is effective in capturing waveform dissimilarity at the extremes of secretion accumulation, it may require further enhancement or combination with additional features to reliably discriminate intermediate secretion levels. To investigate whether patients could be classified based on ventilator waveform changes associated with secretion levels, we applied UMAP, to cluster patients using the features extracted from the VWFs. Figure 7 illustrates the resulting patient clusters, showing clear separation between low and high secretion levels as determined by WSD scores, while medium level exhibits overlap between the two groups. These findings highlight the potential of ventilator waveform analysis for secretion detection and show that the quantified waveform features can be effectively leveraged by machine learning algorithms to enable automated detection and subsequent clustering of patients with similar secretion levels.

### 3.3 Secretion index: A novel metric for airway secretion quantification

A comprehensive assessment of secretion accumulation requires a method that captures its gradual progression and impact on ventilation dynamics. To address this, we have developed a metric that quantifies secretion accumulation by tracking continuous changes in ventilator waveforms over time. Our proposed metric is the Secretion Index (SI) defined as

$$SI=\frac{\left[Q_{\text{max}}+\frac{V_{\text{T}}}{\tau }\right]}{\left[Q_{\text{max0}}+\frac{V_{\text{T0}}}{\tau_{0}}\right]},$$

where $Q_{\text{max}}$ represents the peak inspiratory flow, $V_{\text{T}}$ denotes the tidal volume, and τ is the expiratory time constant. The denominator serves as the baseline reference, which can either be taken as values from a typical healthy adult or from an earlier set of patient measurements. In this study, the baseline is computed from the first recorded breath in the observation window, with $Q_{\text{max0}},V_{\text{T0}},\tau_{0}$ representing the corresponding peak inspiratory flow, tidal volume, and expiratory time constant at that initial time point. The SI represents a scalar metric that integrates airflow dynamics and lung emptying efficiency, offering a comprehensive measure of secretion-related changes in ventilation. The first term, $Q_{\text{max}}$ , serves as an indirect indicator of secretion accumulation, as increased airway secretions are expected to increase airflow resistance, potentially reducing peak inspiratory flow. The second term, $V_{\text{T}}/\tau$ , quantifies how efficiently the tidal volume is expelled relative to airway mechanics, making it sensitive to secretion-induced changes in expiratory flow resistance. Since the expiratory time constant is influenced by both resistance and compliance, this term indirectly captures the impact of secretion-induced airway narrowing and obstruction. A higher SI may indicate efficient secretion clearance, as it suggests robust expiratory flow and effective lung emptying, while a lower SI may signify secretion retention.

**FIGURE 8:**
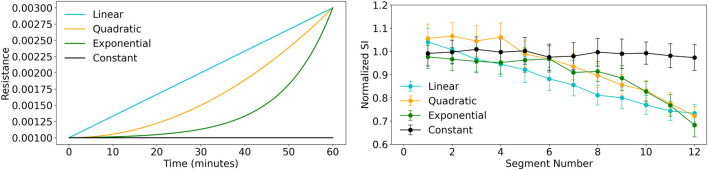
Comparison of resistance conditions and secretion index across segment numbers. Left: Resistance verses time plot shows the resistance progression for different models: Linear (cyan), Quadratic (orange), Exponential (green), and Constant (black). The exponential model shows the fastest growth, while the constant condition remains unchanged. Right: Secretion Index plot shows the median secretion index across 12 segments with standard deviation error bars. The linear model shows the steepest decline, while the constant condition remains stable.

To evaluate the effectiveness of the proposed secretion index, a controlled simulation was designed to generate ventilator waveform data over a 1-h period, corresponding to approximately 720 simulated breaths. The simulation assumed an initially clear airway, setting the resistance parameters $K_{1\text{ETLt}}$ and $K_{1\text{LtLb}}$ to 0.001. Secretion accumulation was then introduced by progressively increasing airway resistance, mimicking secretion accumulation over time. This approach allowed us to examine how the SI evolves as secretion accumulation progressively increases, providing a more comprehensive assessment of secretion-induced changes in ventilation dynamics. Four different resistance evolution patterns were implemented: constant, linear, quadratic, and exponential. In the constant case, resistance remained fixed at the baseline value throughout the simulation, serving as a control. In the linear case, resistance increased gradually from 0.001 to 0.003 across the simulation duration. The quadratic variation simulated a slow initial increase in resistance that accelerated progressively, reflecting a more non-linear accumulation pattern. In the exponential case, resistance remained close to the baseline value for the majority of the simulation, followed by a rapid rise toward the end, reaching a maximum of 0.003. This pattern mimics a sudden obstruction event, with a late-stage, sharp transition to a high-resistance state. The resistance evolution functions were implemented using smooth interpolation schemes, ensuring physiologically plausible transitions between secretion states. Figure 8 (Left) shows a systematic variation in resistance trajectories in this simulation study corresponding to different secretion accumulation scenarios. The SI was computed for each breath and its statistics was analysed across 12 time segments, with each segment representing 60 breaths. Prior to tracking its progression, SI values were normalized by setting the SI of the first breath (clear-airway condition) as the baseline. Correspondingly, the SI exhibited variations following the same trend as airway resistance across different progression profiles (see Figure 8 (Right)). This analysis provides a dynamic perspective on secretion accumulation, complementing the discrete classification approach. This single metric holds potential as a real-time indicator for assessing secretion load and guiding airway clearance strategies, including respiratory physiotherapy, bronchodilator therapy, or suction procedures. Additionally, SI may serve as a valuable parameter for tracking disease progression or assessing treatment response in patients with chronic mucus-related airway diseases.

## 4 Summary and conclusion

This study examined the impact of airway secretions on ventilator dynamics using a mechanistic model combined with patient ventilator waveform data. Simulations were performed to evaluate how secretion accumulation alters respiratory dynamics, demonstrating that increased airway resistance makes it more difficult to mechanically maintain desired ventilation. In particular, increased resistance affects patient-ventilator interactions by reducing peak inspiratory flow and prolonging expiratory time. These effects were reflected in VWF alterations, highlighting the potential for waveform-based analysis to detect secretion accumulation.

To systematically investigate this, we first employed our ventilator model to generate synthetic VWF data under varying airway resistance conditions, corresponding to different secretion levels. The parameters governing airway resistance were selected from different distributions to establish controlled pre- and post-secretion conditions, providing a systematic basis for extracting key waveform features and analysing the impact of secretion removal on ventilator dynamics. Features such as peak inspiratory flow $\left(Q_{\text{max}}\right)$ , tidal volume $\left(V_{\text{T}}\right)$ , and the expiratory time constant (τ) , which were particularly indicative of secretion levels, were identified from this synthetic dataset. To quantify the differences in feature distributions before and after secretion removal, the Wasserstein distance was computed, capturing the degree of change associated with secretion clearance. Building on these insights, we applied the same feature extraction methodology to real patient VWF data, where secretion levels were clinically assessed. Using an unsupervised UMAP clustering algorithm, we observed distinct separation between low and high secretion levels, further demonstrating the potential of VWF-derived markers for non-invasive secretion detection.

Beyond discrete pre/post secretion analysis, we further investigated the continuous progression of airway resistance over time. A 60-min simulation was conducted, where airway resistance gradually increased from a baseline clear-airway state to a high-secretion condition, modelling the dynamic accumulation of secretions. Four different progression profiles were considered: constant, linear, quadratic, and exponential resistance increase. The secretion index, a composite metric derived from ventilator waveform features, consistently tracked the progression of airway resistance in all four cases, demonstrating its potential as a quantitative indicator of secretion accumulation. These findings highlight the potential critical interplay between airway resistance and secretion accumulation, emphasizing the need for timely intervention in response to secretion-induced changes in the ventilator waveform features. Although our model delineates waveform signatures of secretion accumulation, it has certain limitations. It approximates secretion effects solely through resistance changes and does not account for mucus rheology or spatial heterogeneity. The parameter estimation is limited by the lack of additional physiological data (e.g., oesophageal pressure, imaging), which would constrain model parameters and mitigate ill-posedness. While our approach shows promising initial validation on both simulated and clinical data, formal uncertainty quantification (e.g., Bayesian methods) and evaluation in larger patient cohorts are essential to confirm its generalizability and clinical utility. The current model’s translation to diverse patient populations is limited by its simplified representation of airway resistance, without explicitly accounting for condition-specific respiratory mechanics such as compliance variations, lung heterogeneity, or dynamic hyperinflation, as observed in COPD, ARDS, and paediatric cases. Extending the model to such conditions would require incorporation of disease or condition-specific parameters, alongside access to well-labelled ventilator waveform datasets, which remain scarce and present challenges for comprehensive validation. Further studies in these areas will enhance the interpretability and applicability of the framework.

### 4.1 Potential clinical applications and implementation challenges

The proposed mechanistic model and secretion quantification framework holds several practical applications in critical care. Firstly, the secretion index and ventilator waveform features can be used for early detection of airway secretion accumulation, allowing clinicians to initiate suctioning or respiratory physiotherapy before clinical deterioration occurs. Secondly, the model enables real-time monitoring of secretion-related resistance changes, offering the potential for integration into ICU dashboards or ventilator interfaces as a non-invasive indicator of airway patency. This would support timely interventions, particularly in sedated or neurologically-impaired patients. Thirdly, the model-derived metrics could be incorporated into weaning-readiness scores by quantifying secretion accumulation trends, thus improving the prediction of extubation success. Furthermore, personalised adjustment of ventilator settings such as PEEP or pressure support based on inferred resistance and patient effort may reduce ventilator-induced lung injury and improve synchrony. These applications highlight the translational potential of this modelling approach in guiding secretion management, optimizing ventilator strategies, and ultimately enhancing patient outcomes.

Despite its clinical utility, translating this framework into routine ICU practice faces several practical challenges. These include interoperability with existing ICU infrastructure, particularly with ventilator platforms and patient monitoring systems that use diverse data standards and proprietary protocols. Ensuring low-latency data acquisition, secure transmission, and real-time processing will be critical for timely clinical decision support. Robust data storage and management solutions must also be in place to handle the continuous high-frequency waveform data while maintaining patient privacy and compliance with healthcare regulations, such as the European Union General Data Protection Regulation (GDPR) and the U.S. Health Insurance Portability and Accountability Act (HIPAA). Moreover, clinician trust in model-derived indices will depend on transparent validation using real ICU datasets in prospective clinical trials, rigorous performance benchmarking against standard secretion assessment methods, and user-friendly visualization integrated into familiar electronic health record (EHR) interfaces. Comprehensive training and change-management programs will be needed to overcome resistance to adopting novel decision-support tools and to minimize the risk of alarm fatigue (i.e., desensitization caused by excessive non-actionable alarms). Successfully navigating these challenges will pave the way for seamless bedside integration, transforming secretion management and improving patient outcomes in critical care environments.

## Data Availability

The data analyzed in this study is subject to the following licenses/restrictions: The datasets analysed in this study are stored in a secure research environment used for managing sensitive health data. Access to the data can be granted upon reasonable request to the corresponding author. Requests to access these datasets should be directed to n.ovenden@ucl.ac.uk.

## Appendix A

**TABLE A1:** Comparison of model performance for pre- and post-suction breaths across five cases (B1–B5). Metrics include RMSE and $R^{2}$ for airway pressure $\left(P_{aw}\right)$ , airway flow $\left(Q_{aw}\right)$ , and volume (V) .

Breath	RMSE			$R^{2}$		
	$P_{aw}$	$Q_{aw}$	V	$P_{aw}$	$Q_{aw}$	V
Pre-suction						
B1	0.95	147.78	33.50	0.95	0.94	0.95
B2	1.51	77.70	26.43	0.88	0.98	0.97
B3	1.06	152.62	46.31	0.94	0.94	0.91
B4	1.14	177.44	41.81	0.93	0.92	0.93
B5	1.32	85.64	26.85	0.90	0.98	0.97
Post-suction						
B1	1.02	110.20	25.37	0.92	0.96	0.97
B2	1.75	264.60	34.50	0.78	0.74	0.95
B3	1.08	133.57	34.75	0.92	0.93	0.95
B4	1.09	93.02	22.09	0.91	0.97	0.98
B5	2.04	284.33	51.00	0.70	0.70	0.89
